# Antinociceptive and antiedema effects produced in rats by *Brassica oleracea* var. *italica* sprouts involving sulforaphane

**DOI:** 10.1007/s10787-023-01326-6

**Published:** 2023-09-20

**Authors:** Omar Guadarrama-Enríquez, Gabriel Fernando Moreno-Pérez, María Eva González-Trujano, Guadalupe Esther Ángeles-López, Rosa Ventura-Martínez, Irene Díaz-Reval, Agustina Cano-Martínez, Francisco Pellicer, Nieves Baenas, Diego A. Moreno, Cristina García-Viguera

**Affiliations:** 1 Laboratorio de Neurofarmacología de Productos Naturales. Dirección de Investigaciones en Neurociencias, Instituto Nacional de Psiquiatría “Ramón de La Fuente Muñiz”, Calz. México-Xochimilco 101, Col. San Lorenzo Huipulco, 14370 Tlalpan. C.P, Ciudad de Mexico México; 2 Departamento de Farmacología, Facultad de Medicina, Universidad Nacional Autónoma de México, Av. Universidad No. 3000, Col. Ciudad Universitaria, Alcaldía Coyoacán, 04510 Ciudad de Mexico México; 3 Laboratorio de “Farmacología del Dolor” del Centro Universitario de Investigaciones Biomédicas, Universidad de Colima, Av. 25 de Julio 965, 28045 Colima, Col, Mexico; 4 Departamento de Fisiología, Instituto Nacional de Cardiología “Ignacio Chávez”, Juan Badiano 1, Col. Sección XVI, Tlalpan. C.P, 14080 Ciudad de Mexico México; 5 Department of Food Technology, Food Science and Nutrition, Faculty of Veterinary Sciences, Regional Campus of International Excellence “Campus Mare Nostrum”, Biomedical Research Institute of Murcia (IMIB-Arrixaca-UMU), University of Murcia, Espinardo, 30100 Murcia, Spain; 6 Laboratorio de Fitoquímica y Alimentos Saludables (LabFAS), CEBAS, CSIC, Campus de Espinardo - 25, 30100 Murcia, Spain

**Keywords:** Analgesics, Broccoli, Isothiocyanate, Opioids, Sulforaphane

## Abstract

Natural products are recognized as potential analgesics since many of them are part of modern medicine to relieve pain without serious adverse effects. The aim of this study was to investigate the antinociceptive and anti-inflammatory activities of an aqueous extract of *Brassica oleracea* var. *italica* sprouts (AEBS) and one of its main reported bioactive metabolites sulforaphane (SFN). Antinociceptive activity of the AEBS (30, 100, and 300 mg/kg, i.p. or 1000 and 2000 mg/kg, p.o.) and SFN (0.1 mg/kg, i.p.) was evaluated in the plantar test in rats to reinforce its analgesic-like activity at central level using the reference drug tramadol (TR, 50 mg/kg, i.p.). The anti-inflammatory-like response was determined in the carrageenan-induced oedema at the same dosages for comparison with ketorolac (KET, 20 mg/kg, i.p.) or indomethacin (INDO, 20 mg/kg, p.o.). A histological analysis of the swollen paw was included to complement the anti-inflammatory response. Additionally, acute toxicity observed in clinical analgesics as the most common adverse effects, such as sedation and/or gastric damage, was also explored. As a result, central and peripheral action of the AEBS was confirmed using enteral and parenteral administration, in which significant reduction of the nociceptive and inflammatory responses resembled the effects of TR, KET, or INDO, respectively, involving the presence of SFN. No adverse or toxic effects were observed in the presence of the AEBS or SFN. In conclusion, this study supports that *Brassica oleracea* var. *italica* sprouts are a potential source of antinociceptive natural products such as SFN for therapy of pain alone and associated to an inflammation condition.

## Introduction

Nociceptive and inflammatory are the most common types of pain. Nociceptive pain represents a discomforting sensation associated with a real or potential damage of tissue that activates the nociceptors by the noxious stimuli (Woolf 2010). Inflammatory pain occurs after tissue injury and promotes infiltration of immune cells to produce hypersensitivity and repair tissue damage by trauma, heat, and infection (Costigan and Woolf 2000). Inflammation is a biological consequence of protection by vascular tissues against pathogens and irritative agents or damaged cells (Tatiya et al. 2017).

Classifying pain is helpful to guide assessment and treatment. Nonsteroidal anti-inflammatory drugs (NSAIDs) and corticosteroids are used to alleviate pain and inflammation (Eren et al. 2016). Both, NSAIDs and corticosteroids possess disadvantages because of their severe side effects, for example ulcer, osteoporosis, and hepatotoxicity.

Natural products have been studied as potential alternatives of tradicional medicine to alleviate pain and inflammation without adverse effects already reported for conventional analgesic drugs (Ambriz-Pérez et al. 2016). According to the in vivo and in vitro assays, it has been reported that *Brassica oleracea* var. *italica* possesses properties as analgesic, anti-inflammatory, and antioxidant, in part due to chemical compounds called glucosinolates and their derivative products isothiocyanates (Baenas et al. 2017; Guadarrama-Enriquez et al. 2018; Juge et al. 2007), easily converted by gastrointestinal microflora (Fahey et al. 2012), as well as by the presence of phenolic compounds (Ambriz-Pérez et al. 2016), or even vitamins and minerals (Moreno et al. 2006), which improve health benefits of this cruciferous vegetable.

Nutraceuticals are natural products from foods showing relevant properties and benefits in treatment of diseases. *Brassica* genus is of interest for health since the consumption of cruciferous has been associated with anti-inflammatory effects because of the presence of glucosinolates and isothiocyanates, such as the metabolite sulforaphane (SFN) (Fahey et al. 2012; Guadarrama-Enriquez et al. 2018). To reinforce the potential of *Brassica oleraceae* var. i*talica* sprouts as functional food and source of analgesic drugs, in this study was investigated an aqueous extract of this vegetable using enteral and parenteral administration to demonstrate its central and/or peripheral antinociceptive activity and adverse effects or toxicity using experimental models in rats. The effect of SFN alone was also explored as one bioactive metabolite and the involvement of endogenous opioid receptors as its possible mechanism of action.

## Material and methods

### Animals

Male Wistar rats (200–250 g) aged 8 ± 1 weeks were used in this study. The animals were kept at constant room temperature (22 ± 2 °C) and maintained in a 12-h light/dark cycle. All animals were fed ad libitum with standard feed and water. All experimental procedures were carried out according to a protocol approved by local animal bioethics Committee of Instituto Nacional de Psiquiatría Ramón de la Fuente (NC123280.0 and NC17073.0), national [Norma Oficial Mexicana (NOM-062-ZOO-1999)], and international regulations (Zimmermann 1983) to care and use of animal in experimental pain. The rats were manipulated for adaptation to space and experimental conditions at least 3 days before testing.

### Preparation and analysis of the aqueous extract of *Brassica oleracea* var. *italica* sprouts (AEBS)

Seeds of broccoli (*Brassica oleracea* var. *italica*) untreated and ready for sprouting were purchased from Intersemillas S.A. (Valencia Spain). The seeds were washed with 0.5% sodium hypochlorite solution for 2 h and rinsed with Milli-Q type water, followed by aeration for 24 h. The washed seeds were then germinated in a dark chamber at 28 °C for two days, and the germinates were transferred to a growth chamber under controlled conditions (16 h/8 h light/ darkness; 25 °C/ 20 °C air temperature, 60%/80% relative humidity), for six days. Then, sprouts were harvested and freeze-dried (Baenas et al. 2014). The AEBS was prepared as follows: The powder (1.6 g) was resuspended in 50 mL of Milli-Q water and macerated overnight at room temperature. Then, the sample was centrifuged, and the supernatant was filtered and freeze-dried (Baenas et al. 2017). Following the same protocol of Baenas et al. (2017), we analyzed the composition of the broccoli sprouts extract (AEBS) (Table 1).

**Table 1:** Quantification of sulforaphane, glucosinolates and phenolic compounds (mg g-1 d.w.) in broccoli (*Brassica oleracea* var. *italica*) sprouts and the aqueous extract (AEBS)

Bioactive compounds (mg g-1 d.w.)	Broccoli sprouts	AEBS
Sulforaphane	–	0.22 ± 0.051
Glucosinolates (GSL)		
Glucoiberin	5.47 ± 1,10	–
Glucoraphanin	10.94 ± 0.65	–
4-Hydroxyglucobrassicin	1.06 ± 0.08	–
Glucoerucin	2.37 ± 0.18	–
Glucobrasicin	2.92 ± 0.17	–
4-Methoxyglucobrassicin	1.25 ± 0.06	–
Neoglucobrassicin	5.52 ± 0.03	–
Total GSL	29.51 ± 1.42	–
Phenolic compounds		
Chlorogenic acid derivatives	0.40 ± 0.071	0.0044 ± 0.0003
Flavonol glycosides	0.17 ± 0.042	0.0016 ± 0.0006
Sinapic acid derivatives	6.53 ± 1.21	0.031 ± 0.004
Total phenolics	7.10 ± 1.17	0.037 ± 0.004

Mean values (*n* = 3) ± SD. d.w. (dry weight)

### Reagents and drugs

SFN (Cayman Chemical Company Inc. USA), tramadol (TR, AMSA Laboratorios, Mexico), ketorolac (KET, Liomont, S.A. de C.V. Mexico), and indomethacin (INDO, Sigma-Aldrich, St. Louis, MO. USA) were used in this study. All these drugs were prepared with the vehicle (saline solution, 0.9% NaCl). The 1% λ carrageenan (Sigma-Aldrich, St. Louis, MO. USA) was dissolved in saline solution to induce oedema. Oedema was analyzed with Masson’s trichrome stain kit (Hycel^®^).

### Experimental protocol

Different doses of the AEBS using enteral (1000 and 2000 mg/kg, p.o.) or parenteral (30, 100, and 300 mg/kg, i.p.) administration, and a single dosage of SFN (0.1 mg/kg, i.p.), as well as the reference drugs INDO (20 mg/kg, p.o.), KET (20 mg/kg, i.p.), or TR (50 mg/kg, i.p.), were compared to the vehicle groups (p.o. or i.p.) in the nociceptive and inflammatory pain models of the plantar test and the carrageenan-induced oedema test, respectively. All the treatments were explored after 30 min of their administration (Guadarrama-Enríquez et al. 2018).

#### Thermal nociception test

In a preliminary study (Baenas et al. 2017), it was observed that AEBS produced antinociceptive effects in both neurogenic and inflammatory phases of the formalin test. Since the first phase of this test is considered to involve central antinociceptive mechanisms, sixty-six rats were divided into eleven groups of 6 rats per group to receive different doses of the AEBS (30, 100, and 300 mg/kg, i.p. or 1000 and 2000 mg/kg, p.o.) or SFN (0.1 mg/kg, i.p.) and compared to the vehicle groups (i.p. or p.o., respectively) or the reference analgesic drugs INDO 20, p.o., KET 20 or TR 50, i.p., explored in the plantar test. A previous exploratory activity was also tested using the open field test to rule out the possible sedative effects already known for analgesic drugs acting at central nervous system (CNS) as described below.

*Open-field test.* –After 30 min of treatment, the sedative-like response was evaluated by registering ambulatory activity of rats in the open field test. For this, ambulatory activity was tested by placing an individual rat in an acrylic box divided into twelve squares (9 cm × 9 cm). During the experiment, the number of squares explored by each animal with their four limbs was counted during 2 min (Baenas et al. 2017).

*Plantar test.—*Previously to the nociceptive test, rats were acclimated in Plexiglas chambers with heated glass floor for 30 min. Immediately after sedative-like response evaluation, rats were evaluated in the Hargreaves’ apparatus for thermal hyperalgesia. A radiant heat source with a locator light (intensity 60 Hz) was focused under the plantar surface of the right hind paw of the rat until produce a response of shaking or licking. Time of cut of this test was 20 s to prevent tissue damage. The latency of paw withdrawal was determined from an average of 3 separate trails, taken at 3-min interval. Data are expressed as the maximal possible effect (MPE%) calculated as follows (Hargreaves et al 1988; González-Ramírez et al. 2012; Cheah et al. 2017):

$$\mathrm{MPE }\%=\frac{\mathrm{{Latency}\,{treatment}}-\mathrm{{Latency}\, {vehicle}}}{20\mathrm{ s}-\mathrm{{Latency}\, {vehicle}}}\times 100$$

#### Carrageenan-induced rat paw oedema

In a previous study (Baenas et al. 2017), the antinociceptive response of AEBS was reported in a second phase of the formalin test suggesting anti-inflammatory activity. Hence, this effect was reinforced by evaluating antiedema activity *in* vivo using sixty rats which were divided into ten groups of 6 rats per group to receive different doses of the AEBS (30, 100, and 300 mg/kg, i.p. or 1000 and 2000 mg/kg, p.o.) or SFN (0.1 mg/kg, i.p.) and compared to the vehicle groups (i.p. or p.o., respectively) or the reference analgesic drugs INDO 20, p.o., or TR 50, i.p., and tissular damage by histological analysis in this study. The oedema was analyzed in vivo using a vernier caliper, while a histological analysis of the paws of rats, after being euthanized using a CO_2_ chamber, was performance. Stomachs of the rats were also dissected to explore possible gastric damage as it was observed in the presence of NSAIDs like reference drugs.

Thirty minutes after treatment administration, plantar oedema was induced in the subplantar region of the right hind paw of rats with 50 μL 1% λ carrageenan. Inflammation was determined by measuring the thickness of oedema with a vernier caliper at times 0, 1, 2, 3, 4, 5, 6, and 24 h (González et al. 2007). The values were expressed as % of inflammation in the absence or in the presence of the several treatments calculated using the following formula:

$$\mathrm{\% Inflammation }= \frac{\mathrm{{Initial}\,{thickness}}-\mathrm{{Time}\,{thickness}}}{1-\mathrm{{Initial}\,{thickness}}}\times 100$$

*Histology.—*Animals showing significant reduction of oedema in the first 6 h were euthanized, and their hind paws were used for histological analysis. Skin, tarsus, metatarsal, and phalanx bones of paws were removed to obtain only muscle and connective tissues. Tissues were embedded in 30% sucrose at 4 °C for 72 h and after in TissueTek^®^ for 72 h to slice. Tissues were cooled, put-on longitudinal position, and trimmed to obtain slices (thickness 10 μm). Then, tissues were treated with Masson’s trichrome stain (Hycel^®^). They were then mounted on the gelatin microscope slides in the mounting medium. All tissue samples were representative of 4 animals.

*Gastric damage*.—At the end of the test, all animals were euthanized into the CO_2_ chamber, and the stomachs were dissected to examine presence of possible gastric lesions as previously described (Baenas et al. 2017).

#### Acute toxicity

To determine the median lethal dose (LD_50_), the AEBS was given to rats using a preliminary maximal dosage of 2000 mg/kg, by i.p. or p.o. route of administration, according to the OECD guideline for testing of chemicals (2001). After treatment, rats were kept under observation for 14 days to register behavioral changes on ambulatory activity or mortality. Survived rats were euthanized in a CO_2_ chamber, and a general macroscopic observation of tissues was done. If rats died at 2000 mg/kg, the dosage was reduced to 1000 or 100 mg/kg. The parameter of LD_50_ was calculated according to the modified practical method reported by Lorke (1983). In this assay, a minimum number of experimental animals is used to calculate acute toxicity by applying a geometric mean over the doses for which 0% and 100% mortality was found.

### Statistical analysis

Data were expressed as the mean ± standard error of the mean (S.E.M.) of 6 rats as indicated in the figure legends. Area under the curve (AUC) was calculated from the temporal course curves to describe the dose-anti-inflammatory response of the AEBS, SFN, INDO, or KET compared to the vehicle group. Data were analyzed using one or two-way analysis of variance (ANOVA) as appropriate followed by Dunnett’s post hoc test to compare treatments versus control group (vehicle by i.p. or p.o., respectively). A value of *p* < 0.05 was considered significant.

## Results

The broccoli (*Brassica oleracea* var. *italica*) sprouts are rich in glucosinolates, mainly glucoraphanin (Table 1), which is hydrolyzed in the presence of the enzyme myrosinase with maceration in water to obtain the SFN isothiocyanate. The SFN is the reference compound in the AEBS (Guadarrama-Enriquez et al., 2018), and other minor constituents in the AEBS were cinnamic acid derivatives (Table 1).

### Antinociceptive effect of AEBS and SFN in the plantar test

**Fig. 1:**
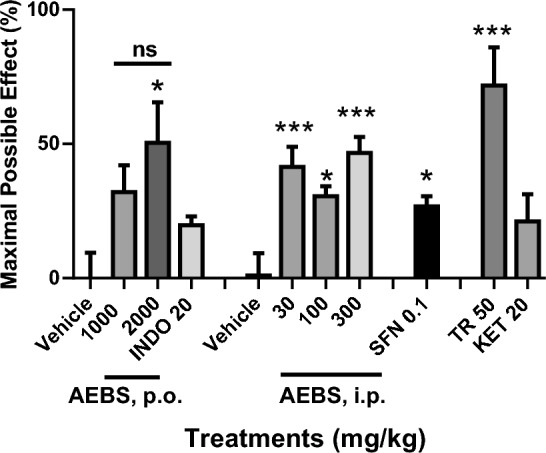
Antinociceptive effects of different doses of the aqueous extract of *Brassica oleracea* var. *italica* sprouts (AEBS) after esophageal (p.o.) or intraperitoneal (i.p.) administration, as well as sulforaphane (SFN) or the analgesic drugs tramadol (TR), ketorolac (KET) or indomethacine (INDO) in comparison to the corresponding vehicle group in the plantar test in rats. Each bar represents the mean ± SEM of six animals. *p < 0.05 and ***p < 0.001, one-way ANOVA followed by Dunnett’s test. *ns*: non-significative

A significant and non-dose-dependent manner analgesic-like response was obtained in the presence of parenteral administration of the AEBS (30, 100 or 300 mg/kg, i.p.) or after SFN at a dosage of 0.1 mg/kg, i.p., reaching 46% and 27% MPE, respectively, compared to the i.p. vehicle group (F_4,25_ = 8.33, *p* = 0.0002). SFN produced an equivalent response than AEBS (100 mg/kg, i.p.) (Fig. 1). Whereas AEBS reached a 32% and 50% MPE after enteral administration of 1000 mg/kg, p.o., or 2000 mg/kg, p.o. (F_2,15_ = 4.697, *p* = 0.026) in comparison to the vehicle group (Fig. 1). A significant MPE of 72% (F_2,15_ = 10.65, *p* = 0.0013) was observed in the presence of the reference drug TR (50 mg/kg, i.p.) but not after administration of KET (20 mg/kg, i.p.) or INDO (20 mg/kg, p.o.) (Fig. 1).

### Anti-inflammatory effects in the carrageenan-induced oedema

**Fig. 2:**
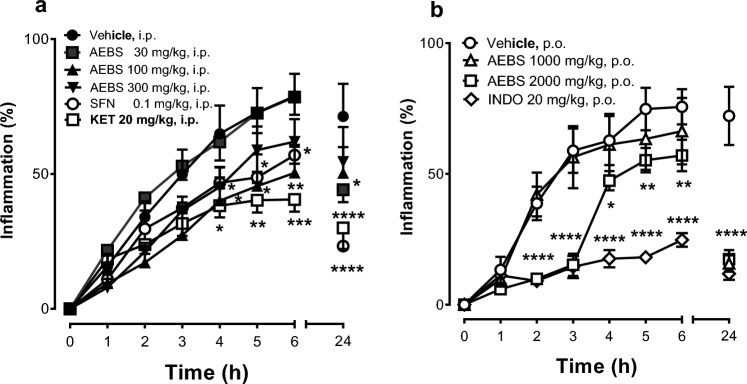
Time course curves (TCC) of the inflammatory response registered each hour during 6 h and then at 24 h using different doses of the aqueous extract of *Brassica oleracea* var. *italica* sprouts (AEBS), sulforaphane (SFN) or the reference analgesic drug ketorolac (KET) in comparison to the vehicle group using intraperitoneal administration (i.p.) in the 1% carrageenan-induced oedema in rats (**a**). As well as the TCC of inflammatory response after different doses of AEBS and the non-antiinflamatory drug indomethacin (INDO) in comparison to the vehicle group using enteral administration (p.o.) (**b**). Each point represents the mean ± SEM of six animals. *p < 0.05, **p < 0.01, ***p < 0.001, or ****p < 0.0001, a two-way ANOVA followed by Dunnett’s test

In the temporal course curves of the experimental evaluation, significant antiedema effects of AEBS were observed after administration of 100 and 300 mg/kg, i.p., or SFN 0.1 mg/kg, i.p., from 4 to 6 h (Fig. 2a), as well as 2000 mg/kg, p.o. from 2 to 6 h (Fig. 2b) resembling the effects of the reference drug KET (20 mg/kg, i.p.) (Fig. 2a) or INDO (20 mg/kg, p.o.) from 2 and 3 h (Fig. 2b). These effects remained until 24 h of inflammation induction. Interestingly, a significant anti-inflammatory response was also reached at lower doses, such as 30 mg/kg, i.p. (Fig. 2a, treatment F_5,255_ = 14.57,* p* < 0.0001; time F_7,255_ = 61.72, *p* < 0.0001; interaction F_35,255_ = 1.586, *p* = 0.0240) or 1000 mg/kg, p.o. (Fig. 2b, treatment F_3,15_ = 12.12, *p* = 0.0003; time F_7,35_ = 95.91,* p* < 0.0001; interaction F_21,105_ = 10.90, *p* < 0.0001).

### Histological analysis

**Fig. 3:**
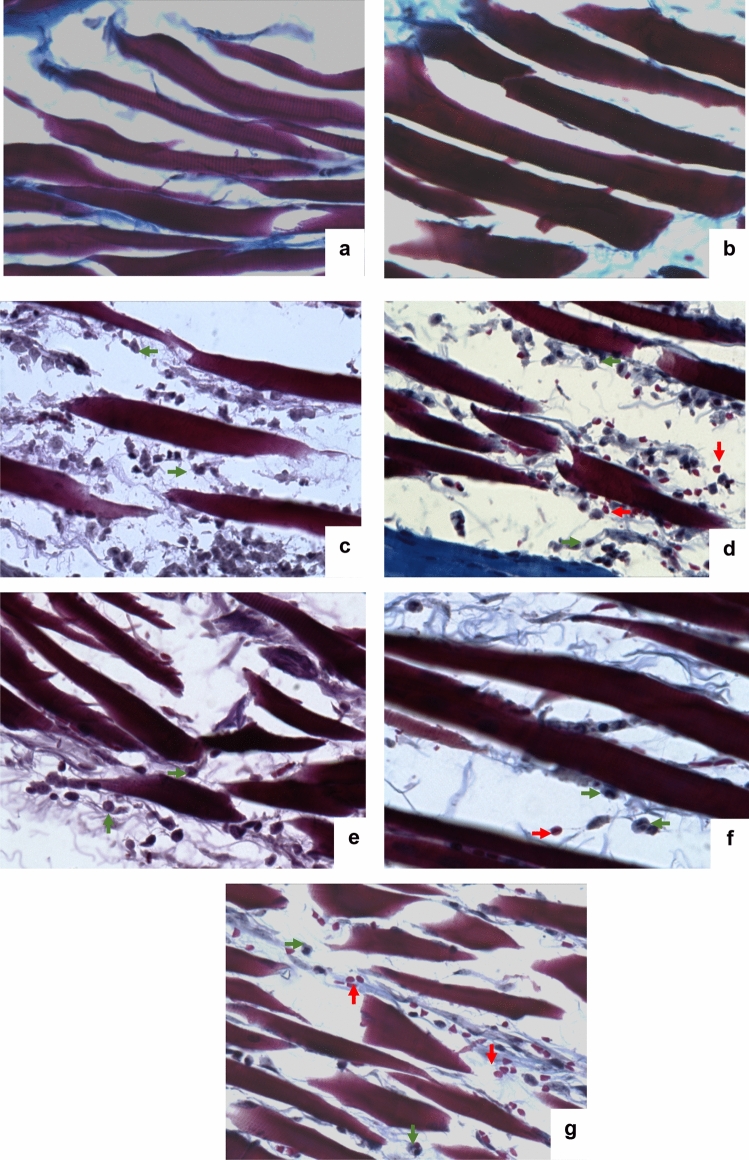
Representative photographs (40x) after Masson’s trichrome stain of the right hind paw tissue of rats from groups: naïve (**a**), saline solution i.p. without oedema (**b**), vehicle (**c**), oedema induced by 1% carrageenan (**d**), aqueous extract of *Brassica oleracea* var. *italica* sprouts (AEBS) at 100 or (**e**), 300 mg/kg, i.p. (f), or ketorolac (KET, 20 mg/kg, i.p.) (**g**). Notice muscular fibers injured showing major separation between fibers with cell infiltration like erythrocytes (red arrows) and leukocytes (orange arrows)

In the histological evaluation, naïve and vehicle groups of rats without oedema demonstrated preserved tissue (Figs. 3a, b, respectively). In contrast, photographs of the inflamed tissue from the paw of rats allowed to observe muscular fibers, oedema, and cellular infiltration in rats receiving intraplantar 1% carrageenan (Figs. 3c, g). Plantar oedema induced with 1% carrageenan in naïve rats or those receiving vehicle alone produced discontinuous fibers and many intrafiber spaces with the presence of abundant cells like erythrocytes, leukocytes, and macrophages (Figs. 3c, d). The presence of the most significant treatment of AEBS at 100 mg/kg, i.p., reduced oedema and cellular infiltration (Fig. 3e), and this response was enhanced when dose was increased to 300 mg/kg, i.p., showing less intrafiber spaces with less cellular infiltration (Fig. 3f). Anti-inflammatory response of AEBS at highest dosage resembled that it was observed in samples from animals receiving the reference drug KET (20 mg/kg, i.p.) (Fig. 3g).

### Adverse effects at central and peripheral level

Sedative-like response is a well-known adverse effect observed in central analgesic drugs such as in opioid therapy. Ambulatory activity of rats was evaluated in the open field in the presence of the vehicle resulting in 26 ± 8 squares explored in 2 min. No alteration on the number of transitions of rats was produced in the presence of the AEBS after enteral administration at 1000 mg/kg, p.o. (23 ± 3) or 2000 mg/kg (26 ± 1) or after INDO 20 mg/kg, p.o. (26 ± 1), neither after parenteral injection at 30 mg/kg, i.p. (30 ± 7), 100 mg/kg, i.p. (20 ± 6), or 300 mg/kg, i.p. (27 ± 11), or in the presence of SFN at 0.1 mg/kg, i.p. (30 ± 5). In contrast, rats treated with TR at 50 mg/kg, i.p., reduced the exploration of rats in a significant manner with a result of 7 ± 6 (F_5,30_ = 4.237, *p* = 0.0049).

**Fig. 4:**
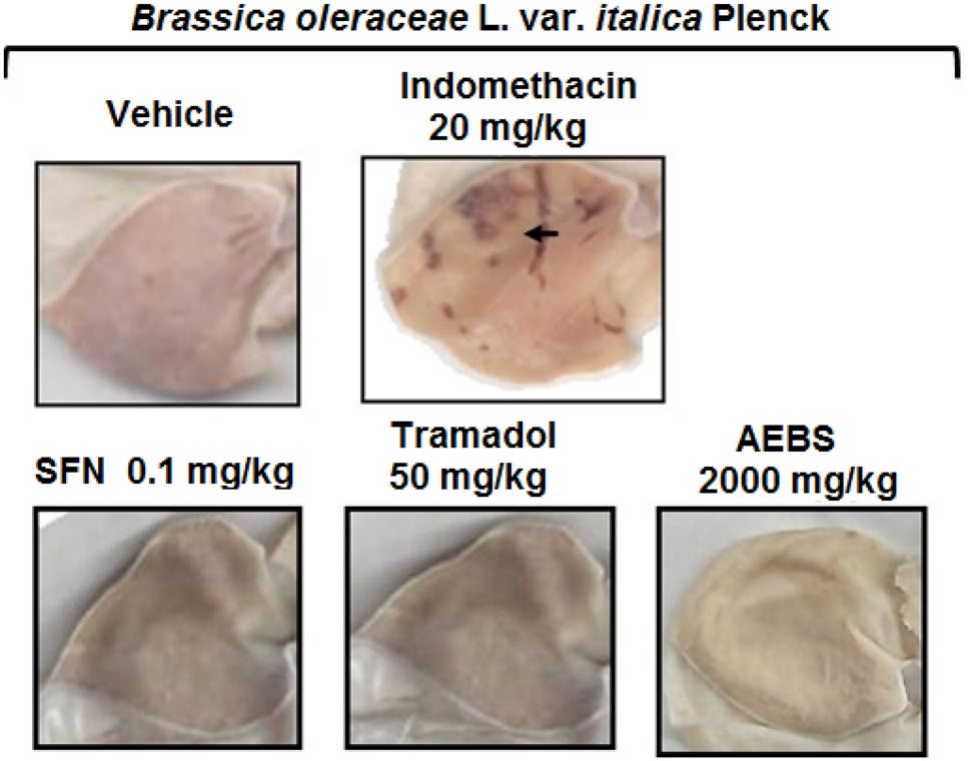
Representative photographs of the rat stomach exploring for gastric damage after administration of tramadol (TR, opioid analgesic at 50 mg/kg, i.p.), sulforaphane (SFN, 0.1 mg/kg, i.p.), and aqueous extract of *Brassica oleracea* var. *italica* sprouts (AEBS) in comparison to indomethacin (INDO, NSAID reference drug, 20 mg/kg, i.p.) as positive control

Rats treated with the AEBS at a dosage of 2000 mg/kg, i.p., SFN (0.1 mg/kg, i.p.), or TR (50 mg/kg, i.p.) were analyzed for gastric damage, but no lesions were produced as compared to those obtained with INDO (20 mg/kg, i.p.) (Fig. 4).

### Acute toxicity

According to the modified Lorke’s method for acute toxicity and calculation of LD_50_, the AEBS showed a value > 2000 mg/kg using esophageal route of administration (p.o.). While using an, i.p., administration, LD_50_ was of 1000 mg/kg.

## Discussion

Different concentrations of metabolites have been found in aqueous extracts depending on the vegetative stage of plants. These differences in the concentration of bioactive metabolites may influence the pharmacological properties of vegetable species as observed in *Brassica oleraceae* var. *italica*, where sprouts showed better antinociceptive response in comparison with seeds and inflorescence (Guadarrama-Enríquez et al. 2018). In our present study, the antinociceptive and anti-inflammatory activities were investigated after enteral and parenteral administration of AEBS corroborating central and peripheral activity in part due to the presence of SFN as one of the bioactive metabolites of this species.

In preliminary studies, the antinociceptive effects of *Brassica oleraceae* var. *italica* inflorescences (Danesh et al. 2014) and sprouts (Baenas et al. 2017) were reported in both phases of the formalin test, a first-choice pain model to screen analgesic drugs (Dubuisson and Dennis 1977; Dzoyem et al. 2017). This test refers two phases: an early (neurogenic) and late (inflammatory) (Tjølsen et al. 1992). In the first phase, nociception is observed in a short period of time due to an involvement of central activity (Lucarini et al. 2013). In the second phase, behavior is more persistent and emphasized due to the tissue damage and inflammatory release mediators (for example: prostaglandins and endoperoxides) (Yamamoto and Nozaki-Taguchi 2002). Preliminary and significant antinociceptive effects of AEBS in both formalin phases in mice suggested central and peripheral activities that were interesting to corroborate in rats using different and complementary experimental models in the present study.

Firstly, AEBS was evaluated in the plantar test, a nociceptive assay using a thermal stimulus to screen analgesics with central action (Sayyah et al. 2004). It is the case of TR, used as a reference analgesic drug in this study, which is a partial opioid agonist that increases the nociceptive threshold in plantar test (Carrillo-Munguía et al. 2015) but produces sedation as an adverse effect (Déciga-Campos et al. 2014). The withdrawal latency of rats increased in all doses of the AEBS tested in the plantar test to reach 50% of the MPE without producing sedative-like effects. It is known that analgesic-like effects of *Brassica oleraceae* var. *italica* sprouts might involve opioid mechanisms (Baenas et al. 2017). Our results support central analgesic-like effects in other *Brassica* species since it was reported that an extract of *Brassica* rapa subspecies chinensis extract increased withdrawal latency in the tail immersion test (Rahman et al. 2015). Preliminary studies have already reported the analgesic-like response of the pure compound SFN in neuropathic (Wang and Wang 2017) and abdominal (Guadarrama-Enriquez et al. 2018) pain, and for the sprouts (Baenas et al. 2017), which effects were avoided in the presence of an opioid antagonist reinforcing the involvement of this inhibitory neurotransmission. The significant antinociceptive effects of the AEBS extract observed in our present study in the plantar test support the involvement of the central action of their components, where the isothyocianate SFN plays an important role since an equivalent antinociceptive response was obtained in its administration alone. In contrast to TR, neither the AEBS (enteral or parenteral administration), nor SFN, produced changes in the ambulatory activity of rats at antinociceptive doses, suggesting the absence of sedative effects.

On the other hand, as previously reported, the AEBS produced antinociceptive effects in the second phase of the formalin test in mice suggesting anti-inflammatory properties (Baenas et al. 2017). To reinforce this activity, the AEBS was evaluated in a well-known and reproducible inflammatory test like carrageenan-induced oedema (Sarkhel 2016). In this test, the acute inflammation is an initial response characterized by induction of ciclooxigenase-2 (COX-2), prostaglandins, blood plasma, and immunologic cells like neutrophils and macrophages that produce reactive oxygen species to modulate inflammatory response in damaged tissue (Tatiya et al. 2017). In our results, the AEBS decreased inflammation resembling the effect of KET. In comparison with the significant antinociceptive response obtained at lowest doses in the plantar test, and the antiedema effect of AEBS was observed at doses of 100 and 300 mg/kg, i.p., or 2000 mg/kg, p.o. suggesting a modulation in the production of inflammatory and vascular prostanoids (Medina et al. 2015). It is known that inhibition of COX-2 is paramount in decrease oedema (Cai et al. 2014). Preliminary reports have already demonstrated that SFN and polyphenols produce anti-inflammatory and anti-edema activities (Yun et al. 2008; Wang & Wang 2017). Differences in SFN bioavailability among ingested forms of *Brassica oleraceae* var. *italica* (raw broccoli or broccoli sprouts) in humans have largely been attributed to differences in myrosinase activity, which is activated by simple chopping or chewing to have relatively high percent recoveries of SFN and SFN metabolites (32–80%) mainly 24 h after consumption (Atwell et al. 2015). In fact, SFN is considered an isothiocyanate at high level of concentrations in *Brassica oleraceae* var. *italica* to produce antioxidant and anti-inflammatory effects through different mechanisms of action including NLRP3 inflammasome inhibition involved in several disorders (Li et al. 2019; Kiser et al. 2021). This might be part of the effects observed after 24 h of the oedema induction not only with SFN but also with the complete AEBS after enteral or parenteral administration in rats in our present study. Our histological analysis demonstrated that AEBS reduced volume of oedema in rats, which was observed as a reduction in the space between muscular fibers with a reduced cellular infiltration like that produced by KET, a NSAID. Anti-inflammatory activity of chemical substances has been studied using in vitro and in vivo assays in tissue injury and infection associated to chronic inflammation. Mechanism for this activity includes enzymatic inhibition on the nitric oxide synthase (NOS), COX-1, and COX-2 and pro-inflammatory factors as tumoral necrosis factor (TNF-α) and interleukin-1 (IL-1β) through inactivation of NF-κB (Heiss et al. 2001; Rotelli et al 2003; dos Santos et al. 2006; Yun et al. 2008). These mechanisms have been reported for anti-inflammatory activity of SFN to decrease pain behavior in gout and neuropathic pain, but also in traumatic spinal cord injury by inhibition of NF-κB, interleukins IL-1β, IL-6, and TNF-α (Wang and Wang 2017). At peripheral level, inhibition of some of these inflammatory mediators might be part of the anti-edema mechanism of the AEBS since SFN is one active metabolite.

Physiologically, COX-1 catalyzes prostaglandins for gastric and renal protection; a common mechanism of action for NSAIDs is the inhibition of COX isoenzymes producing severe gastric injury (Frölich 1997). To this respect, the AEBS or SFN alone did not produce gastric damage as compared to the reference NSAID indomethacin (INDO, 20 mg/kg, i.p. or p.o.). It has been reported that pro-inflammatory interleukins and COX-2 have been inhibited by polyphenols and sulfur compounds contained in species of genus *Brassica* (Rotelli et al. 2003; dos Santos et al. 2006; Yun et al. 2008) suggesting that these mediators might cause less gastric damage in contrast to the COX-1 involvement. It is important to mention that AEBS did not produce toxicity or lethal effects at doses producing antinociceptive and anti-inflammatory activities in rats.

The LD_50_ was calculated of 1000 mg/kg by single i.p. administration and doses greater than 2000 mg/kg by esophageal route. Oral administration of an ethanol extract using the leaves of *Brassica oleracea* var. *italica* and chronic administration for 28 consecutive days neither produced toxicity (Shah et al. 2016). According to the global harmonized system (GHS) classification, the results of acute toxicity of AEBS explored after enteral or parenteral administration in this study classified them in category 5 or as minimal or slight hazard, standard categories for health products (OEDC, 2001).

In the future, it will be important to explore non-sedative effects of high doses of AEBS in a simultaneous electroencephalographic analysis and other bioactive metabolites of *Brassica oleracea* var. *italica* that were limitations of this study.

## Conclusion

*Brassica oleracea* var. *italica* produces antinociceptive and anti-edema activities involving the presence of SFN as partial responsible. Commonly adverse effects of modern analgesics are omitted in the activity of *Brassica oleracea* var. *italica* sprouts and SFN suggesting its potential as functional food and source of new drugs for pain therapy.

## Data Availability

Data supporting the findings of this study are available from the corresponding author [M.E.G.T.] on request.

## Abbreviations

AEBS: Aqueous extract of *Brassica oleracea* var. *italica* sprouts
ANOVA: Analysis of variance
AUC: Area under the curve
CNS: Central nervous system
COX: Cyclooxygenase
INDO: Indomethacine
IL: Interleukin
KET: Ketorolac
LD_50_: Median lethal dose
NF-κB: Nuclear factor kappa B
NOS: Nitric-oxide synthase
NSAID: Nonsteroidal anti-inflammatory drugs
SEM: Standard error of the mean
SFN: Sulforaphane
TNF-α: Tumor Necrosis factor-α
TR: Tramadol
