# Editorial Expression of Concern: Antinociceptive and antiedema effects produced in rats by *Brassica oleracea* var. *italica* sprouts involving sulforaphane

**DOI:** 10.1007/s10787-025-02050-z

**Published:** 2025-12-01

**Authors:** Omar Guadarrama-Enríquez, Gabriel Fernando Moreno-Pérez, María Eva González-Trujano, Guadalupe Esther Ángeles-López, Rosa Ventura-Martínez, Irene Díaz‑Reval, Agustina Cano-Martínez, Francisco Pellicer, Nieves Baenas, Diego A. Moreno, Cristina García-Viguera

**Affiliations:** 1 Laboratorio de Neurofarmacología de Productos Naturales, Dirección de Investigaciones en Neurociencias, Instituto Nacional de Psiquiatría “Ramón de La Fuente Muñiz”, Calz. México-Xochimilco 101, Col. San Lorenzo Huipulco, 14370 Tlalpan. C.P, Ciudad de Mexico, Mexico; 2 Departamento de Farmacología, Facultad de Medicina, Universidad Nacional Autónoma de México, Av. Universidad No. 3000, Col. Ciudad Universitaria, Alcaldía Coyoacán, 04510 Ciudad de Mexico, Mexico; 3 Laboratorio de “Farmacología del Dolor” del Centro Universitario de Investigaciones Biomédicas, Universidad de Colima, Av. 25 de Julio 965, 28045 Colima, Col, Mexico; 4 Departamento de Fisiología, Instituto Nacional de Cardiología “Ignacio Chávez”, Juan Badiano 1, Col. Sección XVI, Tlalpan. C.P 14080, Ciudad de Mexico, México; 5 Department of Food Technology, Food Scienceand Nutrition, Faculty of Veterinary Sciences, Regional Campus of International Excellence “Campus Mare Nostrum”, Biomedical Research Institute of Murcia (IMIB-Arrixaca-UMU), University of Murcia, Espinardo, 30100 SciencesMurcia, Spain; 6 Laboratorio de Fitoquímica y Alimentos Saludables (LabFAS), CEBAS, CSIC, Campus de Espinardo - 25, 30100 Murcia, Spain

**Expression of Concern to: Inflammopharmacology (2023) 31:3217–3226**

10.1007/s10787-023-01326-6

The Editor-in-Chief would like to alert the readers that concerns have been raised about Fig. 4, SNF 0.1 mg/kg and Fig. 4, Tramadol 50 mg/kg, as they appear to be highly similar.

The authors have stated that the duplication occurred accidentally while preparing the figure. However, upon request they were not able to provide the original raw images.

Readers are therefore advised to interpret these results with caution.

Irene Díaz-Reval disagrees with this Editorial Expression of Concern.

Omar Guadarrama-Enríquez, Gabriel Fernando Moreno-Pérez, María Eva González-Trujano, Guadalupe Esther Ángeles-López, Rosa Ventura-Martínez, Agustina Cano-Martínez, Francisco Pellicer, Nieves Baenas, Diego A. Moreno and Cristina García-Viguera did not respond to correspondence from the Publisher about this Editorial Expression of Concern.

